# Association of NGF and Mitochondrial Respiration with Autism Spectrum Disorder

**DOI:** 10.3390/ijms231911917

**Published:** 2022-10-07

**Authors:** Maria Gevezova, Danail Minchev, Iliana Pacheva, Tatyana Todorova, Ralitsa Yordanova, Elena Timova, Ivan Ivanov, Victoria Sarafian

**Affiliations:** 1 Department of Medical Biology, Medical University-Plovdiv, 15 A Vasil Aprilov Blvd, 4000 Plovdiv, Bulgaria; 2 Research Institute at Medical University-Plovdiv, 4000 Plovdiv, Bulgaria; 3 Department of Pediatrics and Medical Genetics, Medical University-Plovdiv, 4000 Plovdiv, Bulgaria; 4 Clinic of Pediatrics, St. George University Hospital, 4000 Plovdiv, Bulgaria

**Keywords:** ASD, mitochondrial function, NGF, Seahorse XFp

## Abstract

Background: NGF is a molecule with a pleiotropic role, affecting neuro-immune functions, energy homeostasis, and synaptic plasticity. The mechanisms of NGF-induced neuronal differentiation are well established, but its effect on mitochondria in autism spectrum disorder (ASD) is still unclear. We hypothesize that NGF-induced neuronal development requires large amounts of energy, and mitochondria in ASD are overloaded to meet the new functional requirements. Method: The study includes primary diagnosed ASD children. Peripheral blood mononuclear cells (PBMCs) and plasma were obtained from both patients and typically developing children (TDC). PBMCs were analyzed with Seahorse XFp, and plasma NGF protein levels were measured. Results: We detected nearly 50% higher NGF levels and approximately 40% elevation in spare respiratory capacity in ASD compared to TDC. These findings are consistent with the observed difference in maximal respiration, which was also significantly higher in the patient group. Both mitochondrial respiration and NGF plasma levels exhibit a strong potential to discriminate children with ASD from TDC. Conclusions: This study is the first to link elevated NGF with mitochondrial respiration and altered energy homeostasis in ASD. High NGF correlates with basic bioenergetic signatures that may be used as a screening tool to improve early diagnosis and clinical follow-up in ASD.

## 1. Introduction

Autism spectrum disorder (ASD) is a psychiatric impairment that presents in early childhood with persistent deficits in social communication and interaction, and restricted, repetitive patterns of behavior, interests, or activities. In the past, autism was thought to be a purely psychological or neurological disorder. Current laboratory and clinical evidence suggest increased oxidative stress, mitochondrial dysfunction, and inflammation in a significant proportion of idiopathic ASD patients. They affect mainly energy-dependent cells such as neurons, gastrointestinal and immune cells [1,2,3,4,5,6].

In addition, recent data suggest the involvement of nerve growth factor (NGF) in the pathogenesis of ASD [7,8]. This pleiotropic molecule performs a number of activities related to the following functions:

(1) modelling of neurodevelopment, neuroplasticity and maintenance of the functional integrity of cholinergic neurons in the central nervous system (CNS) [9];

(2) regulation of the concentration of reduced glutathione (GSH) via the MEK/MAP signaling pathway [10];

(3) remodeling of mitochondrial function and maintenance of cell energy homeostasis [11,12,13]. The location of NGF receptors (TrkA and p75) in the inner mitochondrial membrane supports its direct role in mitochondrial biogenesis [14,15];

(4) regulation of the immune response in inflammation.

**Figure 1:**
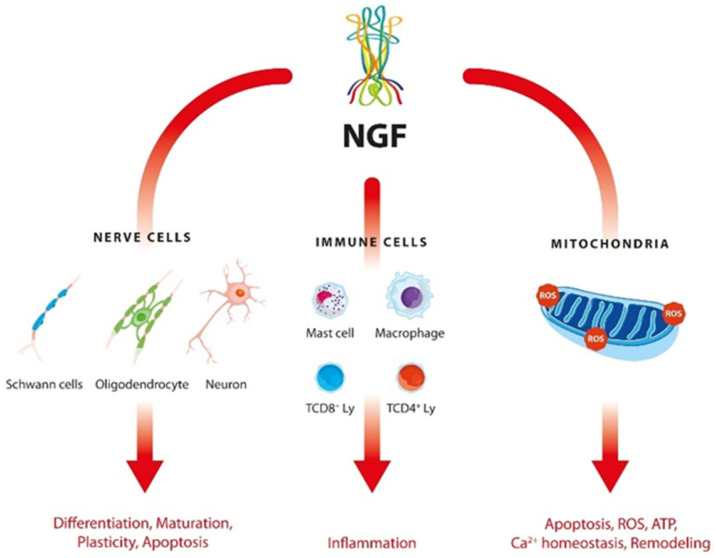
Interactions of NGF with nerve and immune cells in ASD.

The complex interactions between NGF and nerve and immune cells are shown in Figure 1.

Despite the limited and contradictory data on NGF levels in children with ASD, a meta-analysis revealed significantly higher values in peripheral blood of affected individuals than in healthy controls [7]. A study by Liu et al. (2021) provides clinical evidence for its key role in the development of ASD [7]. Other authors even suggest NGF as a serological marker of ASD [14,16]. It is supposed that, in ASD patients, abnormal expression of neurotrophic factors occurs during embryogenesis, and it continues into adulthood [17]. This may explain the structural alterations in the brains of ASD children. Magnetic resonance imaging (MRI) studies show that abnormal brain growth is observed up to 2 years of age [18,19,20,21,22]. From 2 to 4 years, excessive growth is followed by pathological deceleration or even ceases [23,24]. These data suggest that the neuronal development and synaptic plasticity are not occurring in a typical pattern [25,26,27,28]. Additionally, in patients with ASD, smaller neurons and dendritic branches, fewer Purkinje cells, and excessive neuronal production have been revealed in different brain areas [25,26,27,28]. Chronically activated microglia and abnormal neuroinflammation are also found [1,29,30].

Mitochondria play an important role in neurogenesis, providing energy for synaptic function and axonal growth [31,32]. A number of studies have reported that neuronal differentiation is associated with metabolic reprogramming [33,34,35,36], increased mitochondrial biogenesis [33], mitophagy [34,37] and oxidative stress [38,39,40]. Armijo-Weingart et al. (2019) confirmed that NGF induces mitochondrial division and subsequent maintenance of stable state of these organelles in embryonic sensory axons [15]. NGF-dependent regulation is considered as a mandatory component for the subsequent branching of axons [15]. These observations paved the way for further investigations on the role of this neurotrophin in mitochondrial function and in regulation of nerve cell survival, branching, axon regeneration, and synaptic function. The balance change of the mitochondrial division/fusion equilibrium contributes to dysfunction of these organelles and is a cornerstone for the cell adaptation to energy needs, cell recovery or cell death [41,42,43,44].

There is growing genetic, clinical, and biochemical data indicating mitochondrial dysfunction as one of the most common metabolic abnormalities in ASD [2,5,45,46]. Increased levels of lactate, glutathione and intermediates from the Krebs cycle, ATP deficiency, and decreased activity of mitochondrial electron transport chain [46,47,48,49] in blood samples, cerebrospinal fluid and brain tissue from patients with ASD are found [1,5,50]. In addition, in our previous study on children with ASD, we have discovered mitochondrial dysfunction, altered adaptive response to oxidative stress, and strong dependence on fatty acids in ASD patients [1]. In this context, we suppose that ASD may involve disorders in various systems, not just the CNS.

The expression of neurotrophic factors and mitochondrial activity are interrelated because the differentiation of neurons requires a large amount of energy and “building blocks”. This in turn alters biochemical and energetic processes in mitochondria which strive to meet the higher ATP consumption and the new functional requirements [12]. The mechanisms of NGF-induced neuronal differentiation are revealed, but there is limited knowledge about its effect on mitochondrial function, especially in various pathologies.

We hypothesize that NGF-induced neuronal development requires large amounts of energy, and mitochondria in ASD appear to be overloaded to meet the new functional necessities. To test this hypothesis, we examined protein levels of NGF in parallel with mitochondrial activity in ASD patients and healthy controls. Our results present novel evidence for altered mitochondrial function and its correlation with NGF in children with ASD.

## 2. Results

### 2.1. Clinical Data

Following extensive medical examination, 14 children from the target group were identified as having regression. The ADOS scores were as follows: communication—5.89 (±1.8); social interaction—13.97 (±3.7); play—5.15 (±1.2); repetitive behavior—6.06 (±1.8). ADOS raw score was 25.93 (±7.1), and the IQ was calculated as 50.16 (±14.1).

### 2.2. Detection of NGF Plasma Levels

**Figure 2:**
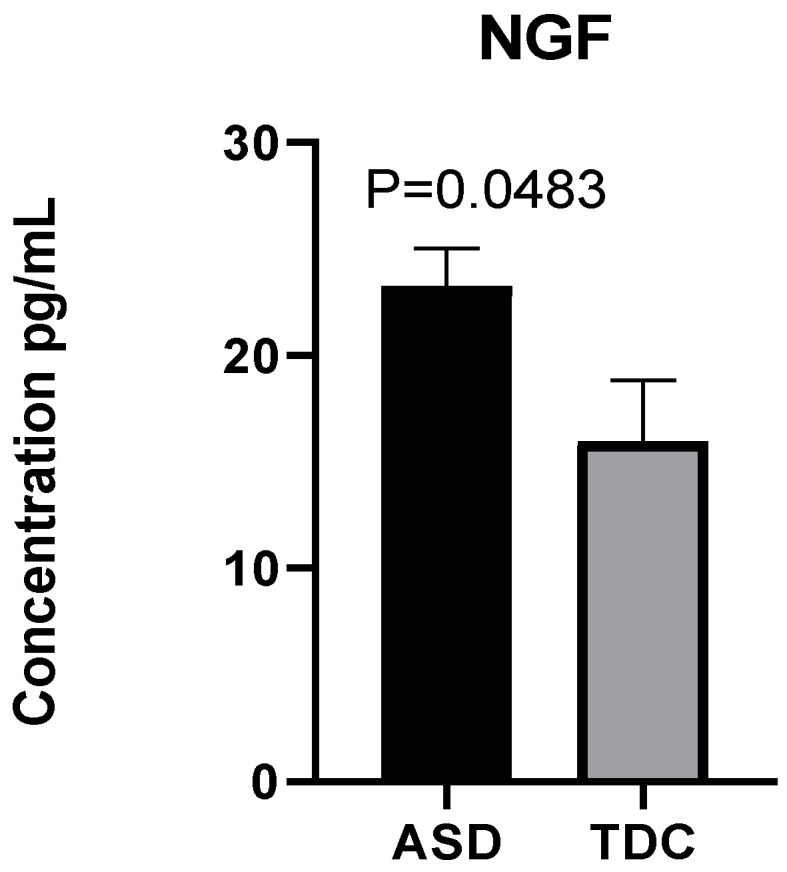
Elevated plasma NGF in children with ASD (*n* = 40) compared to typically developing children (TDC) (*n* = 12).

Our results show that plasma levels of NGF in ASD children are nearly 50% higher than those in typically developing ones. An arithmetic mean of 23.25 pg/mL was reported in children with ASD and 15.98 pg/mL in the control group. A parametric Welch’s *t*-test indicates that the observed difference is statistically significant: Welch’s t (14.82) = 2.15, *p* = 0.0483 (Figure 2).

### 2.3. Respiratory Profile in Children with ASD and Healthy Controls

#### 2.3.1. Mito Stress Test–Oxygen Consumption (OCR)

To assess the mitochondrial function of PBMCs isolated from children with ASD and TDC, we used an extracellular flux analyzer to determine the rate of oxygen consumption (OCR) and the degree of extracellular acidification (ECAR; Seahorse Bioscience). Seahorse XFp analyzer evaluates four main parameters: (1) Basal respiration; (2) Produced ATP; (3) Proton leak; and (4) Maximal respiratory capacity.

**Figure 3:**
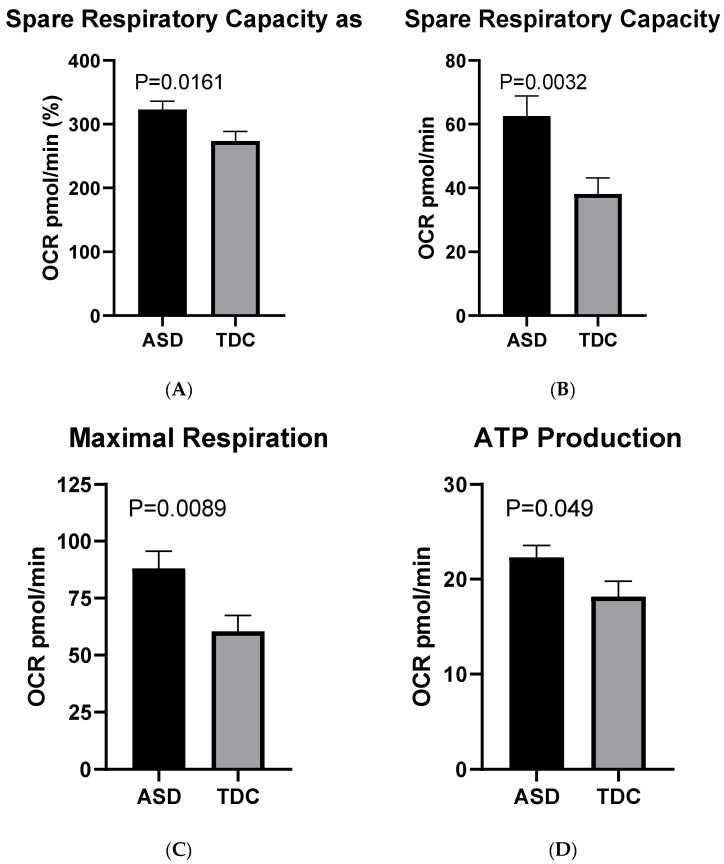
Basic respiratory parameters in ASD (*n* = 40) and TDC (*n* = 12): (**A**) spare respiratory capacity as %; (**B**) spare respiratory capacity as absolute values; (**C**) maximal respiration; (**D**) ATP production.

The bioenergetic measurements showed a statistically valid difference in the Spare Respiratory Capacity percentage between the two groups of interest. On the average, this particular respiratory parameter was approximately 18% higher in PBMCs of children with ASD (arithmetic mean = 323%), compared to healthy controls (273%): Welch’s t (61.77) = 2.47, *p* < 0.016 (Figure 3A).

When the Spare Capacity was measured in absolute values without prior normalization, the disparity between the ASD group (Mean = 62.60 pmol/min) and the control one (Mean = 38.19 pmol/min) appeared even more prominent (approximately 61%). The indicated difference is considered significant with Welch’s t (91.47) = 3.03, *p* = 0.003 (Figure 3B).

These findings were concordant with the observed variation in the Maximal Respiration, which was prominently higher in the ASD (Mean = 88.05 pmol/min) group compared to the control (60.48 pmol/min): Welch’s t (76.67) = 2.68, *p* = 0.009 (Figure 3C).

Finally, another respiratory parameter—ATP production showed only a moderate difference between the two examined groups: Mean (ASD) = 22.30 pmol/min and Mean (TDC) = 18.15 pmol/min; Welch’s t (51.64) = 2.02, *p* = 0.048 (Figure 3D). In contrast, no statistically significant alterations between the two groups in terms of Basal Respiration, Proton Leak, and Coupling Efficiency were detected. The lack of statistical difference in basal respiratory rates indicates a fairly uniform initial bioenergetic state in both ASD and control PBMCs, which in turn supports the reliability of the measurements performed.

#### 2.3.2. Mito Stress Test-Extracellular Acidification (ECAR)

Simultaneously with the measurement of OCR, the Seahorse XFp analyzer also evaluates the rate of extracellular acidification (ECAR) of the culture medium in which PBMCs are incubated. This is an indirect method for analyzing the glycolytic rates of cells.

**Figure 4:**
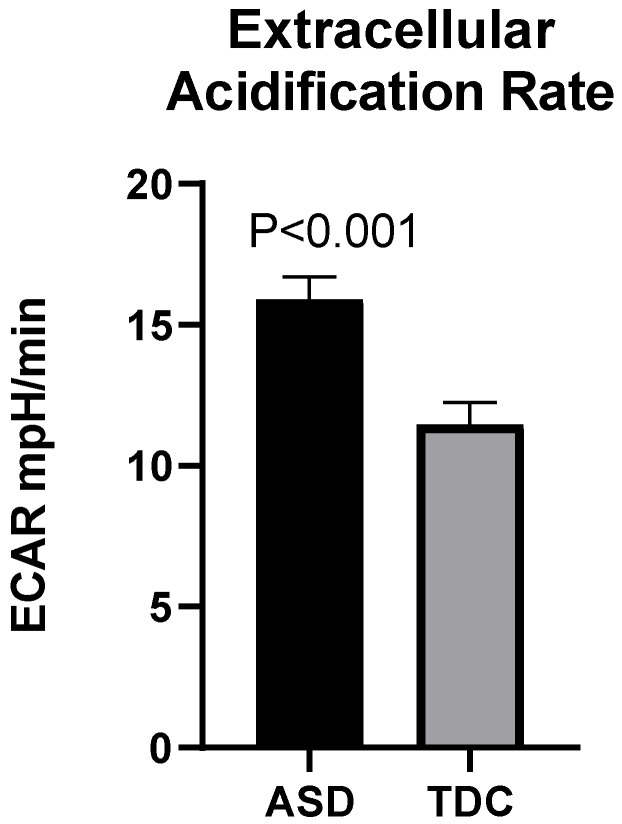
Increased extracellular acidification (ECAR) in children with ASD (*n* = 40) compared to TDC (*n* = 12).

The observed differences in mitochondrial respiration parameters were accompanied by enhanced glycolytic activity in the ASD group, as demonstrated by the ECAR. At baseline, the average ECAR levels in the ASD group (15.90 mpH/min) showed a tendency of increase compared to those in the control group (11.46 mpH/min) which is statistically significant [Welh’s t (72.175) = 3.98, *p* < 0.001] (Figure 4).

Following the addition of mitochondrial inhibitors during the Mito Stress experiment, the difference observed between the two groups retained its statistical validity (Supplementary Figure S1).

### 2.4. Correlation between Plasma Levels of NGF and Mitochondrial Respiration

In the overall cohort of ASD and TDC, we observed a moderate positive correlation between the plasma levels of NGF and the following parameters of mitochondrial respiration, shown in Table 1.

We detected no correlation between NGF levels and other parameters like Basal Respiration, ATP Production, Spare Respiratory Capacity, and Coupling efficiency (*p* > 0.1).

**Figure 5:**
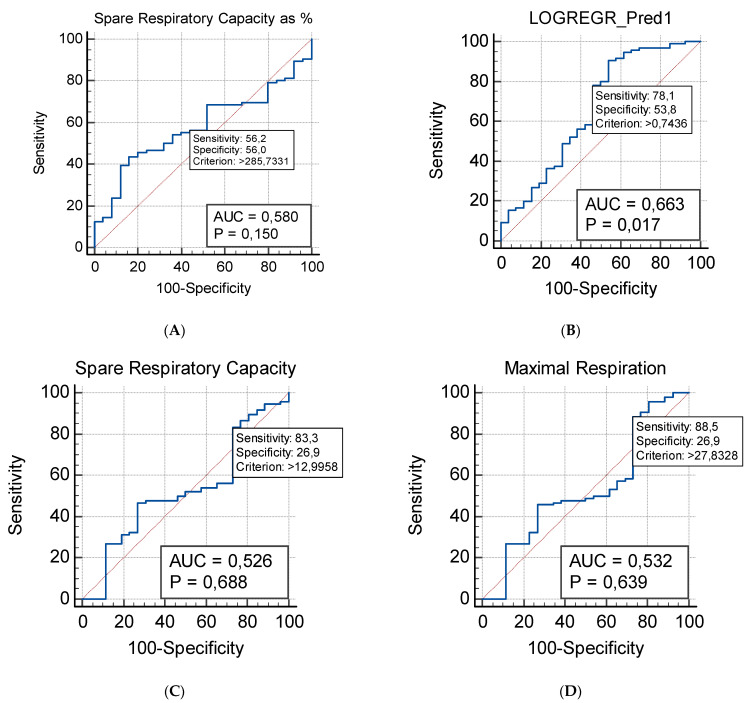
ROC (Receiver Operating Characteristic) analysis of mitochondrial respiration parameters in patients with ASD (*n* = 40) (**A**) Spare Respiratory Capacity as %; (**B**) Additive model that involve the Maximal Respiration and the Spare Respiratory Capacity; (**C**) Spare Respiratory Capacity as absolute values; (**D**) Maximal Respiration.

To investigate the role of mitochondrial respiration parameters and the plasma levels of NGF as potential diagnostic markers, we performed an ROC (Receiver Operating Characteristic) analysis of the experimental data (Figure 5).

The Spare Capacity is the mitochondrial respiratory parameter exhibiting the most prominent difference between the studied groups. It shows the strongest predictive capability as a potential diagnostic biomarker: AUC = 0.58, 95% CI (0.487–0.670), Sensitivity = 0.562, Specificity = 0.560 (Figure 5A). When an additive ROC model was established, it used the predicted probabilities, previously assessed by a logistic regression analysis. Indeed, the additive model that combines the Maximal Respiration and the Spare Respiratory Capacity exhibited the strongest predictive capabilities (Figure 5B). The additive ROC discriminated ASD children from healthy controls more accurately than any of the respective parameters individually (evident from the ROC curves in Figure 5C,D).

A logistic regression approach was used to evaluate the possible association between the obtained parameters of mitochondrial respiration and ASD. Our regression model suggests significant contributions of both the Maximal Respiration and the Spare Respiratory capacity. The model provides a better fit than the null hypothesis with χ^2^ = 9.848 and *p* = 0.007 and results in the following logistic coefficients (Beta weights): for the Constant: B =2.7925; SE: 0.6089; Wald = 21.0366; *p* < 0.001; for the Maximal Respiration predictor variable: B = −0.1106; SE = 0.03873; Wald = 8.1498; *p* = 0.004; for the Spare Respiratory Capacity: B = 0.1345; SE: 0.04769; Wald = 7.9486; *p* = 0.004. The estimated odds ratio indicates a decrease of approximately 10% [Exp (B) = 0.8953, 95% CI (0.8299–0.9659)] for ASD for every unit increase of Maximal Respiration. The estimated odds ratio indicates an increase of approximately 14% [Exp (B) = 1.1439, 95% CI (1.0418–1.2560)] for ASD for every unit increase of Spare Respiratory Capacity.

The present study suggests no statistically valid association between patients’ age and gender, neither between plasma levels of NGF and the indicators of mitochondrial respiration. No correlation was found between NGF levels and any of the clinical parameters assessed on admission—ADOS score, presence or absence of developmental regression, DQ/IQ. No significant association was identified between any of the respiratory parameters and the DQ/IQ, or the ADOS scores of the examined ASD children. However, our experimental data demonstrated a strong association between the presence of developmental regression and two of the evaluated parameters of mitochondrial respiration (Spare Respiratory Capacity as % and Coupling Efficiency). The Spare Capacity was almost 40% higher in PBMCs of ASD children with developmental regression (Mean = 383%) compared to those without regression (Mean = 274%). The observed difference was statistically significant (Welch’s t (45.4) = 3.54, *p*-value < 0.001). Furthermore, PBMCs of ASD children with regression exhibited a significantly lower Coupling Efficiency (Mean = 77.2%) compared to those of children without regression (Mean = 82.5%; Welch’s t (45.4)).

**Figure 6:**
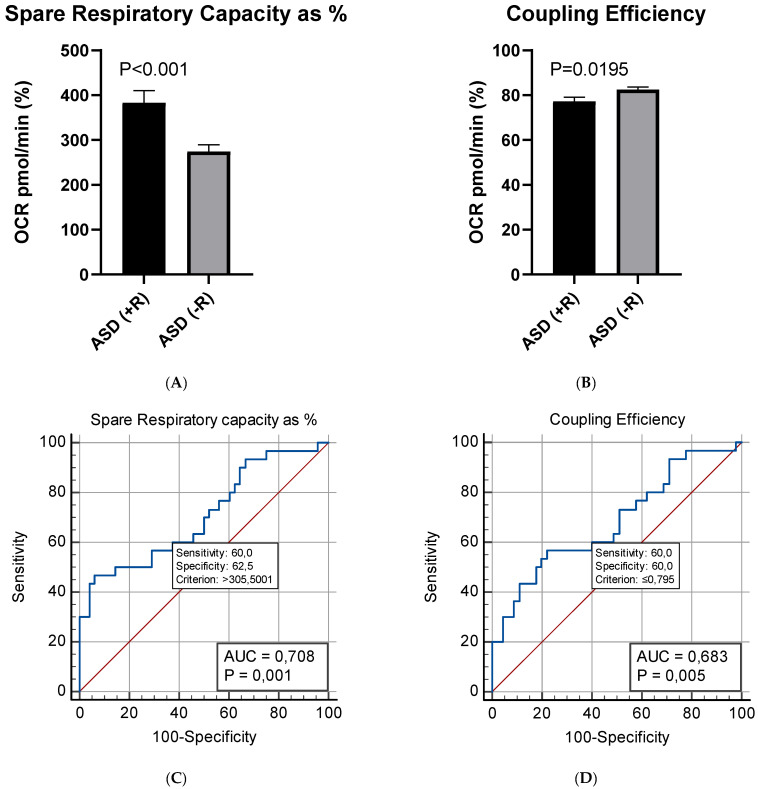
Mean differences in: (**A**) Spare Respiratory Capacity as %; (**B**) Coupling Efficiency between children with (+R) (*n* = 15) or without (−R) (*n* = 25) developmental regression; (**C**,**D**) ROC analysis of mitochondrial respiration parameters in children with and without developmental regression.

The subsequent ROC analysis demonstrated that the two respiration parameters may presumably serve as putative bioenergetics markers that reflect the presence or absence of developmental regression in children with ASD (Figure 6).

## 3. Discussion

The present study demonstrates statistically valid differences in NGF levels and mitochondrial function between children with ASD and TDC. It also examines potential associations between the studied mitochondrial respiration indicators, NGF, and clinical manifestations of the disease. We report a prospective correlation between NGF and bioenergetic changes in patients with ASD compared to the control group. Our experimental results show that plasma NGF levels in children with autism (arithmetic mean = 23.25) are approximately 50% higher than those in TDC (arithmetic mean = 15.98). To date, scientific data on the levels of neurotrophic factors in children with ASD remain contradictory. However, our results are consistent with a previous meta-analysis by Liu et al. (2021) that demonstrates that NGF is markedly overexpressed in blood samples from ASD children [7]. In this study, the authors have linked the significant overexpression of NGF with the previously known abnormalities in brain development accompanying patients with ASD [7,20,51]. Furthermore, NGF has been revealed as an essential modulator with complex pleiotropic action, affecting neuro-immune functions, oxidative homeostasis and leading to a change in the balance between cells and developmental signal pathways in the CNS [52,53,54].

In addition, the expression of NGF receptors on the mitochondrial membrane suggests its role in the regulation of biogenesis of these organelles. NGF is believed to ensure an endogenous defense mechanism against the swelling of mitochondria induced by intracellular Ca^2+^ influx in mitochondria [12,14,55]. Two major NGF signal induction pathways control mitochondrial morphogenesis—PI3K and Mek-Erk [15]. They trigger mitochondrial division, which is a mandatory component of NGF-induced axon branching (via the TrkA receptor) [56]. It in turn activates PI3K which regulates Drp1-dependent mitochondrial division [15]. NGF-mediated modulation of mitochondrial function is important for neurogenesis, nerve cell regeneration, and energy supply. Moreover, NGF was long ago shown to stimulate B-lymphocyte growth and differentiation, which extends its activity far beyond the CNS [57].

Altered mitochondrial biogenesis by NGF and abnormal mitochondrial dynamics have been implicated in the etiology and progression of ASD, as well as some of the more common neurological diseases associated with mitochondrial dysfunction [58]. These observations prompted us to investigate the relationship between NGF and mitochondrial respiration to measure the rate of oxygen consumption (OCR) in PBMCs isolated from patients and healthy controls. Using the Mito Stress Test, we found the following key differences in mitochondrial function: increased respiratory reserve capacity (in both percentage and absolute values), maximal respiration, and ATP production in the patient group. The observed differences in the reported parameters may potentially reflect higher mitochondrial oxidative phosphorylation PBMCs of ASD children. As shown previously, high values of reserve respiratory capacity may reflect adaptation to oxidative stress [59]. However, this is often associated with higher sensitivity to ROS in patient samples and the inability to respond to the increased energy demands of cells [1]. Mitochondrial disfunction and oxidative stress have also been demonstrated in postmortem patients’ brain regions related to speech, auditory processing, motor coordination, social behavior and memory [45,60,61]. In addition, NGF participates in the regulation of ROS defense mechanisms and maintains oxidant homeostasis by stimulating antioxidant enzymes (such as GSH/GSH-Px redox cycle, superoxide dismutase, glutathione peroxidase, glutathione reductase, catalase, etc.) and glutathione metabolism [62,63,64].

On the other hand, the lack of a marked average difference in ATP production in our ASD cohort can be explained by the significant in-group variance of this particular parameter for both PBMC populations. This observation is consistent with a previous study in which NGF-treated cells display enhanced, although not significant, ATP-linked respiration six hours after treatment [12]. In search of insights into the effects of NGF on mitochondrial function, we examined correlations with Seahorse-derived parameters.

We found positive correlations between the plasma levels of NGF and the respiratory parameters Maximal Respiration and Proton Leak (which may reflect increased activity of the NADPH oxidase and the production of ROS). These results are concordant with previous studies in cell lines (PC12) showing an NGF-dependent mechanism leading to mitochondrial remodeling [12,65,66] involving accelerated metabolism [35,36] and biogenesis [12,33,67] in order to clear the depleted mitochondria [12,36,68,69]. According to Martorana et al. (2018), NGF-induced mitochondrial remodeling in a PC12-615 cell line involves enhanced basal respiration, maximal respiration, proton leak, and spare capacity without a significant change in ATP production [12]. Of particular interest is the fact that proton leak is the only parameter that changes prominently six hours after NGF treatment in vitro [12]. This is the very same variable we found to correlate most strongly with NGF levels in plasma. Furthermore, it has been largely discussed that proton leak is not only a sign of damage but also a regulatory mechanism. Elevated ROS levels stimulate proton leak, while proton leak decreases the accumulation of ROS [70].

We also report an increased rate of extracellular acidification (ECAR) in PBMCs isolated from patients, which indirectly indicates a boost in glycolytic processes and non-mitochondrial respiration. These findings may be a potential consequence of the intensified metabolism in PBMCs from ASD children. Our data are supported by the results obtained in positron emission tomography (PET) and magnetic resonance imaging (MRI) in children with ASD [71,72,73]. PET studies revealed an augmented rate of glucose uptake in the primary visual cortex (calcarine fissure) and hypometabolism in the temporal areas (anterior cingulum, putamen, and thalamus) [71]. These results suggest that ASD involves systemic metabolic disorders that affect all cells. In this context, the changes we report can give a valuable insight into the regulation of brain metabolism provided that neuroactive mediators in the systemic circulation also affect PBMCs. Generally, the data obtained from our study show for the first time that elevated NGF in patients with ASD is correlated with two parameters of mitochondrial function and altered energy homeostasis.

The modulation of mitochondrial activity by NGF may be important for neuro regeneration or neurogenesis, although it is not clear yet whether these changes are a result or a consequence of ASD.

However, it is widely recognized that the immune system and the CNS are interconnected on many functional levels. Almost 60% of active genes marked by H3K4me3 are common to both neurons and PBMCs [74]. Numerous cytokine receptors are expressed in the CNS. Moreover, messenger RNA for interleukin-2 and T-cell receptors have been found in neurons [75]. Lymphocytes express several receptors for neurotransmitters and hormones, including dopamine, acetylcholine and serotonin receptors, as well as receptors for glucocorticoid and mineralocorticoid hormones [76]. There is solid evidence that lymphocytes can be directly affected by glucocorticoids and catecholamines [77]. In line with these findings, peripheral blood can provide valuable markers of the clinical state of the organism, even when it comes to processes not directly related to the functions of the blood itself [78]. Bioenergetic markers in peripheral blood have been previously studied in the context of neurological or psychiatric conditions such as: Parkinson’s disease [79], amyotrophic lateral sclerosis [80], Huntington’s disease [81], and major depression [82].

To further explore the association between mitochondrial function and the abnormalities observed in ASD, we looked for a correlation with some clinical characteristics. We describe a significant correlation between the reserve respiratory capacity and the coupling efficiency, and the development of regression in our patient group. This allows us to suspect the potential role of mitochondria in developmental regression in ASD children. Furthermore, we found that reserve respiratory capacity is almost 40% higher in ASD children with developmental regression compared to those without, which is regarded by some authors as a protective adaptation in response to environmental stressors [83]. Alternatively, a possible explanation of the reported lower coupling efficiency (the ratio between the oxygen consumption used for ATP synthesis and the one leading to proton leakage) in regressed children may be provided by the so-called “leak metabolism” hypothesis [84]. Licznerski et al. (2020) proposed an “immature” type of metabolism in which ATP is produced primarily by aerobic glycolysis rather than by mitochondrial oxidative phosphorylation [84]. The authors also attribute the cause of this phenotype to a disruption of the mitochondrial inner membrane associated with ATP production (abnormal levels of the c-subunit of the ATP synthase), leading to an abnormal delay in neuronal development. An interesting finding is the fact that closure of the leak allowed synapse maturation and normalized ASD behaviors [84].

From another perspective, previous data demonstrating increased serum NGF levels in ASD children showed NGF levels to correlate with hyperserotonemia [8]. Heightened serotonin levels have long been associated with ASD [85]. Recent work indicates that the raised serotonin levels in ASD may arise from a failure of serotonin to be used as a necessary precursor for the melatonergic pathway [86]. This may arise as a consequence of the inhibition of 14-3-3, which is necessary to stabilize arylalkylamine N-acetyltransferase (AANAT), as well as from a decrease in acetyl-CoA availability as a necessary co-substrate for AANAT [86]. This could suggest that the alterations in mitochondrial function in ASD may be arising from a decrease in the capacity to induce the mitochondrial melatonergic pathway, leading to the loss of multiple melatonin effects on mitochondrial function and consequently on cellular, including immune cell, function [86]. A number of microRNAs (miRNAs) can inhibit 14-3-3 and the melatonergic pathway, including miR-375 and miR-451, with miR-451 shown to be increased in the platelets, intestinal epithelial cells and pinealocytes in ASD [87]. As to whether these or other miRNAs underpin mitochondrial dysfunction coupled to raised levels of NGF and serotonin will be important to determine in future research.

It has also been demonstrated that ASD-related metabolic signatures may also be associated with the plethora of data showing alterations in the gut microbiome and gut permeability in ASD [88]. Interestingly, recent data show that indole-3-acetate, via aryl hydrocarbon receptor (AhR) activation, increases NGF in neuronal cell lines [89]. The AhR shows some inhibitory effects on 14-3-3 in different cell types, whilst the AhR-induced cytochrome P450 (CYP)1b1 can backward convert melatonin to N-acetylserotonin (NAS), which is a brain-derived neurotrophic factor (BDNF) mimic via its activation of the BDNF receptor TrkB [90]. Given that such neurotrophic receptor activation can lead to the induction of other neurotrophins [91], it requires clarification as to whether the upregulation of the NAS/melatonin ratio contributes to both suboptimal mitochondrial function and increased NGF levels. The proposed suppression of the melatonergic pathway will also have significant consequences regarding the beneficial effects of the gut microbiome-derived short-chain fatty acid, butyrate, which increases mitochondria-located sirtuin-3, leading to the deacetylation and disinhibition of the pyruvate dehydrogenase complex (PDC), which converts pyruvate to acetyl-CoA, thereby optimizing mitochondrial function and being permissive of melatonergic pathway induction. As such, butyrate’s many beneficial effects may be partly dependent upon the capacity of cells to upregulate the melatonergic pathway [92]. This could also suggest that decreased butyrate in ASD may be contributing to decreased mitochondrial function and increased NGF, as well as wider alterations in epigenetic processes. Future research will be required to determine the relevance of these associations.

A significant limitation of the present study is in the inability to distinguish whether the increased NGF concentrations were due to proNGF (activating apoptotic pathways) or to mature forms of NGF (differentiative/survival effect), but we certainly detected its effect on mitochondrial function in children with ASD. In addition, the observed elevated plasma NGF levels and mitochondrial changes in PBMCs may not reflect strictly those in the CNS. Another limiting feature of this study is its relatively small sample size, which potentially narrows its statistical power. Nevertheless, the studied groups include a sufficient number of probands for the respective conclusions to be made.

## 4. Materials and Methods

### 4.1. Subjects

Forty Bulgarian children with ASD aged 2 to 11 years and a control group of 12 typically developing children (TDC) matched for age and sex were included in the study. All subjects were examined by a team of a pediatric neurologist, psychologist, and child psychiatrist in the Department of Pediatrics, St. George University Hospital, Plovdiv. ASD was suspected by clinical exam and proven by an Autism Diagnostic Observation Schedule (ADOS) [93]. Developmental regression, found in 14 in ASD patients, was defined as a loss of at least one developmental achievement (i.e., combining 2–3 words into sentences) that persists more than 3 months and is not explained by another illness (i.e., hypacusis). Developmental quotient (DQ) or intelligence quotient (IQ) were determined. The Wechsler Intelligence Scale for Children Fourth Edition (WISC IV) for children over 6 years of age and Developmental Profile-3 (DP-3) for children under 6 years of age were used [94,95] (Supplementary Table S1).

Cases of syndromic autism were excluded after clinical, radiological, and metabolic investigations. Patients with additional chronic or acute illnesses, epileptic seizures, or taking medications or supplements such as vitamins, cofactors, and immunomodulators were also excluded from the study. After completing the informed written consent procedure, blood samples were taken by vein puncture in EDTA-Vacutainer monovette (S-Monovette 2.6 mL, Z-Sarstedt).

TDC and ASD children were tested on different days as each of them had a personal appointment of clinical and laboratory tests.

The study was approved by the Ethics Committee at Medical University of Plovdiv (Protocol No C-05-2/10.04.2020).

### 4.2. Isolation of Plasma

After centrifugation for 10 min at 1800 rpm, plasma from patients and healthy controls was carefully separated and aliquoted into sterile tubes and then stored at –80 °C prior to further analysis.

### 4.3. Isolation of Peripheral Blood Mononuclear Cells (PBMCs)

PBMCs were processed immediately after collection within 2 h, which limits the negative effect of freezing. They were isolated using Pancoll (Pan Biotech Cat № P04-60500) according to the manufacturer’s protocol. In brief, the blood was mixed with 2 mL of phosphate buffered saline (PBS) (pH 7.4.) and pipetted onto Pancoll in a 1:1 ratio. Then, it was centrifuged for 30 min at 400× *g* with minimal acceleration and deceleration. The buffy coat containing PBMCs was aspirated into a new 15 mL Falcon tube and washed twice with 10 mL PBS, centrifuged at 250× *g* for 10 min. Cells were cultured in RPMI-1640 medium (Pan Biotech Cat # P04-22100) supplemented with 10% FBS, and 1% penicillin/streptomycin. Cells were grown in an incubator overnight at 37 °C, 5% CO_2_, and high humidity. The number of cells and their viability were determined using an automated counter “LUNA” (Logos Biosystems, Anyang, Korea). Cells were diluted with RPMI-1640 to a final concentration of 2 × 10^5^ cells per well and then placed in 8-well Seahorse microplates for metabolic analysis. Cell viability was measured by counting the number of living cells and plating exactly 2 × 10^5^ living PBMCs per each well for the subsequent bioenergetic tests, since PBMCs are sensitive and die out easily cell counting and viability checks were always done immediately before plating (which was immediately followed by acquisition of the bioenergetic data).

### 4.4. NGF Detection by ELISA

Plasma samples were analyzed in duplicate for NGF levels using the Human NGF ELISA Kit (ELISA Genie, Dublin, Ireland, Cat No. D62XSQSEP7), following the manufacturer’s instructions.

### 4.5. Mito Stress Test

Mitochondrial OCR was determined by the most innovative technology to date to study mitochondrial activity—the Seahorse XFpanalyzer (Agilent, Santa Clara, CA, USA). Cells were seeded at a density of 2 × 10^5^ cells per well in Seahorse XFp base medium pH 7.4. To ensure that PBMCs were evenly distributed in the wells (in one layer), they were visualized using an inverted microscope before analysis. Each sample was tested in triplicate (total number of experiments *n* = 52), and the results were averaged. Mitochondrial function was measured after injection of the following inhibitors: oligomycin, FCCP and rotenone according to the manufacturer’s instructions (Table 2).

### 4.6. Statistics

Statistical analyses were performed with GraphPad Prism v.9.2.0 (GraphPad Software Inc., San Diego, CA, USA), MedCalc v.20.010 (MedCalc Software, Ostend, Belgium), and R v.3.6.1. R: A language and environment for statistical computing. R Foundation for Statistical Computing, Vienna, Austria.). Differences between normally distributed variables were evaluated for significance using a Welch’s t-test for independent samples. For non-normal data, the non-parametric Wilcoxon–Mann–Whitney assessment was preferred. The significance threshold was set at a *p*-value < 0.05. Data obtained from Seahorse (Bioscience, Agilent) were analyzed using Wave software.

## 5. Conclusions

A correlation between NGF levels and mitochondrial respiration in children with ASD is detected. As neural differentiation requires large amounts of energy and “building blocks”, mitochondria in ASD appear to be overloaded and depleted to meet the new growing functional requirements.

The changes detected in mitochondrial respiratory parameters and NGF concentrations may provide a better understanding of the pathophysiology of ASD and serve as a screening tool to improve the early diagnosis and clinical follow-up of patients with ASD.

## Figures and Tables

**Table 1:** Correlation between plasma levels of NGF and mitochondrial respiration.

Mitochondrial Parameters	Pearson’s r	Pearson’s *p*
Maximal Respiration	r (35) = 0.30	*p* = 0.075
Proton Leak	r (36) = 0.34	*p* = 0.038

**Table 2:** Inhibitors of mitochondrial function for Mito Stress Test.

Inhibitor	Final Concentration	Function
Oligomycin	1.5 µM	ATP synthase inhibitor
FCCP	2 µM	Stimulated OCR
Rotenone	0.5 µM	ETC complex I inhibitor

## Data Availability

The data presented in this study are available in the article.

## Supplementary Materials

The following supporting information can be downloaded at: https://www.mdpi.com/article/10.3390/ijms231911917/s1, Figure S1: Oxygen consumption rates and extracellular acidification rates of patients with ASD and TDC; Table S1: Demographic and clinical characteristics of patients with ASD.
