# Supplementary material for: Association of NGF and Mitochondrial Respiration with Autism Spectrum Disorder
Source: Int J Mol Sci. 2022 Oct 7;23(19):11917. doi: 10.3390/ijms231911917 (PMC9569874; doi:10.3390/ijms231911917)

Supplementary Figure S1. Oxygen consumption rates and extracellular acidification rates of patients with ASD and TDC.

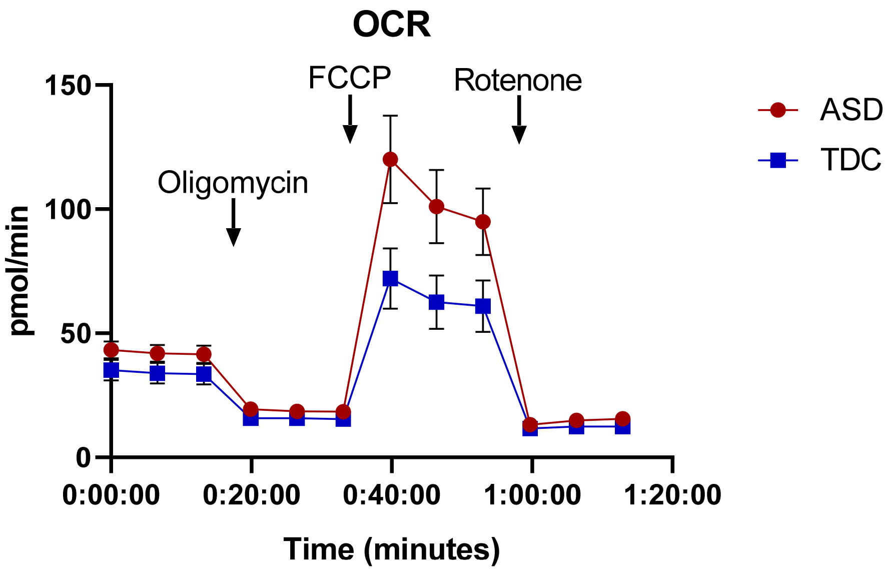

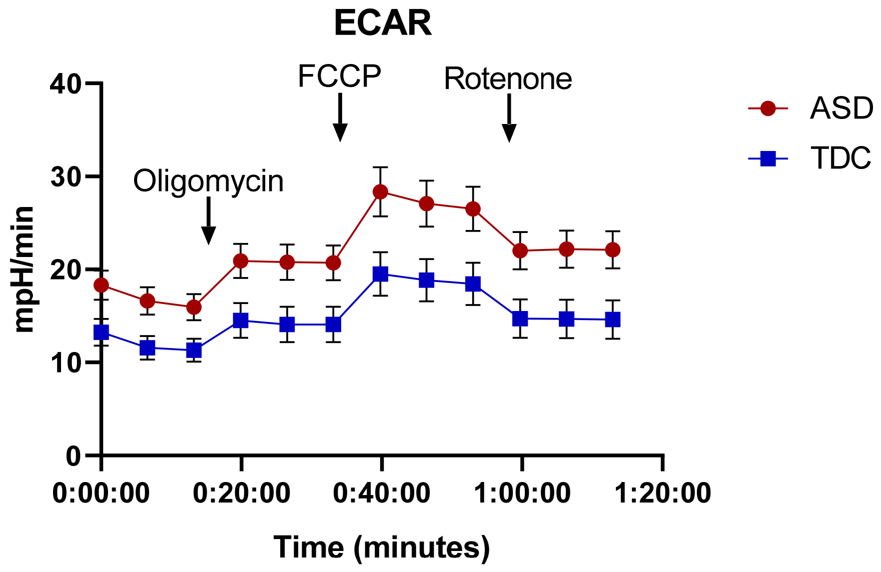

Supplement: Supplementary file 1 [file ijms-23-11917-s001.zip › Supplementary Figure S1 (Gevezova et al).pdf]
