# Supplementary material for: Association of NGF and Mitochondrial Respiration with Autism Spectrum Disorder
Source: Int J Mol Sci. 2022 Oct 7;23(19):11917. doi: 10.3390/ijms231911917 (PMC9569874; doi:10.3390/ijms231911917)
Supplement: Supplementary file 1 [file ijms-23-11917-s001.zip › Supplementary Table S1 (Gevezova et al).pdf]

Supplementary Table S1. Demographic and clinical characteristics of patients with ASD.

Target groups	Number of participants	Age (years)	Sex		Number of ASD with regression	ADOS					IQ
			M	F		Commu-nication	Social interaction	Play	Repetitive behavior	ADOS raw score	
ASD	40	2-11	35	5	15	5.89 (±1.8)	13.97 (±3.7)	5.15 (±1.2)	6.06 (±1.8)	25.93 (±7.1)	50.16 (±14.1)
TDC	12	2-12	8	4	-	-	-	-	-	-	-
